# Association of Fluid Balance With Short- and Long-term Respiratory Outcomes in Extremely Premature Neonates: A Secondary Analysis of a Randomized Clinical Trial

**DOI:** 10.1001/jamanetworkopen.2022.48826

**Published:** 2022-12-29

**Authors:** Michelle C. Starr, Russell Griffin, Katja M. Gist, Jeffrey L. Segar, Rupesh Raina, Ronnie Guillet, Saudamini Nesargi, Shina Menon, Nekayla Anderson, David J. Askenazi, David T. Selewski

**Affiliations:** 1 Division of Nephrology, Department of Pediatrics, Indiana University School of Medicine, Indianapolis; 2 Pediatric and Adolescent Comparative Effectiveness Research, Department of Pediatrics, Indiana University School of Medicine, Indianapolis; 3 Department of Epidemiology, University of Alabama at Birmingham, Birmingham; 4 Division of Cardiology, Department of Pediatrics, Cincinnati Children’s Hospital Medical Center, University of Cincinnati, Cincinnati, Ohio; 5 Division of Neonatology, Departments of Pediatrics and Physiology, Medical College of Wisconsin, Milwaukee; 6 Department of Nephrology, Akron Children's Hospital, Akron, Ohio; 7 Division of Neonatology, Department of Pediatrics, Golisano Children's Hospital, University of Rochester, Rochester, New York; 8 Department of Neonatology, St Johns Medical College Hospital, Bangalore, Karnataka, India; 9 Division of Nephrology, University of Washington and Seattle Children's Hospital, Seattle; 10 Division of Nephrology, Department of Pediatrics, University of Alabama at Birmingham, Birmingham; 11 Division of Nephrology, Department of Pediatrics, Medical University of South Carolina, Charleston

## Abstract

**Importance:**

Extremely low gestational age neonates are at risk of disorders of fluid balance (FB), defined as change in fluid weight over a specific period. Few data exist on the association between FB and respiratory outcomes in this population.

**Objective:**

To describe FB patterns and evaluate the association of FB with respiratory outcomes in a cohort of extremely low gestational age neonates.

**Design, Setting, and Participants:**

This study is a secondary analysis of the Preterm Erythropoietin Neuroprotection Trial (PENUT), a phase 3 placebo-controlled randomized clinical trial of erythropoietin in extremely premature neonates conducted in 30 neonatal intensive care units in the US from December 1, 2013, to September 31, 2016. This analysis included 874 extremely premature neonates born at 24 to 27 weeks’ gestation who were enrolled in the PENUT study. Secondary analysis was performed in November 2021.

**Exposures:**

Primary exposure was peak FB during the first 14 postnatal days. The FB was calculated as percent change in weight from birth weight (BW) as a surrogate for FB.

**Main Outcomes and Measures:**

The primary outcome was mechanical ventilation on postnatal day 14. The secondary outcome was a composite of severe bronchopulmonary dysplasia (BPD) or death.

**Results:**

A total of 874 neonates (449 [51.4%] male; mean [SD] BW, 801 [188] g; 187 [21.4%] Hispanic, 676 [77.3%] non-Hispanic, and 11 [1.3%] of unknown ethnicity; 226 [25.9%] Black, 569 [65.1%] White, 51 [5.8%] of other race, and 28 [3.2%] of unknown race) were included in this analysis. Of these 874 neonates, 458 (52.4%) received mechanical ventilation on postnatal day 14, and 291 (33.3%) had severe BPD or had died. Median peak positive FB was 11% (IQR, 4%-20%), occurring on postnatal day 13 (IQR, 9-14). A total of 93 (10.6%) never decreased below their BW. Neonates requiring mechanical ventilation at postnatal day 14 had a higher peak FB compared with those who did not require mechanical ventilation (15% above BW vs 8% above BW, *P* < .001). On postnatal day 3, neonates requiring mechanical ventilation were more likely to have a higher FB (5% below BW vs 8% below BW, *P* < .001). The median time to return to BW was shorter in neonates who received mechanical ventilation (7 vs 8 days, *P* < .001) and those with severe BPD (7 vs 8 days, *P* < .001). After adjusting for confounding variables, for every 10% increase in peak FB during the first 14 postnatal days, there was 103% increased odds of receiving mechanical ventilation at postnatal day 14 (adjusted odds ratio, 2.03; 95% CI, 1.64-2.51).

**Conclusions and Relevance:**

In this secondary analysis of a randomized clinical trial, peak FB was associated with mechanical ventilation on postnatal day 14 and severe BPD or death. Fluid balance in the first 3 postnatal days and time to return to BW may be potential targets to help guide management and improve respiratory outcomes.

**Trial Registration:**

ClinicalTrials.gov Identifier: NCT01378273

## Key Points

**Question:**

Is fluid balance associated with respiratory outcomes in extremely premature neonates?

**Findings:**

In this secondary analysis of a placebo-controlled randomized clinical trial in 874 premature neonates, fluid balance during the first 2 postnatal weeks was associated with mechanical ventilation and bronchopulmonary dysplasia. The time to return to birth weight was shorter in neonates who continued to receive mechanical ventilation, and every 5% increase in fluid balance was associated with an increase in the odds of mechanical ventilation.

**Meaning:**

In premature neonates, fluid balance and more rapid return to birth weight were associated with mechanical ventilation and bronchopulmonary dysplasia.

## Teaser

This secondary analysis of a randomized clinical trial of neonates examines whether a more positive fluid balance during the first 2 postnatal weeks is associated with mechanical ventilation on postnatal day 14.

## Introduction

Extremely low gestational age neonates (ELGANs) commonly develop multiorgan dysfunction, which frequently includes disordered fluid balance.^1^ The impact of disordered fluid balance and, at its extreme, fluid overload on morbidity and mortality is an increasing area of focus in neonatal and pediatric critical care.^1,2,3,4,5,6,7^ An important step in improving outcomes in the care of ELGANs is to better understand how fluid balance impacts distant organs and patient outcomes.^8^ A complete understanding of the role of fluid balance on the duration of mechanical ventilation in ELGANs is critical because this represents a potential target for intervention to improve outcomes.

Fluid overload occurs commonly in preterm neonates and is associated with increased morbidity and mortality.^9^ Using data from the Assessment of Worldwide Acute Kidney Injury Epidemiology in Neonates (AWAKEN) study,^1^ we showed that positive fluid balance in the first postnatal week is associated with mechanical ventilation at postnatal day 7 in neonates born at less than 36 weeks’ gestational age. Additionally, studies^10,11,12^ have shown that fluid overload is associated with the development of bronchopulmonary dysplasia (BPD). The epidemiology and impact of fluid overload on short- and long-term respiratory outcomes in ELGANs has not been evaluated in a multicenter study.

The Preterm Erythropoietin Neuroprotection Trial (PENUT) study^13,14,15^ captured robust data on fluid balance, kidney-related outcomes, and duration of mechanical ventilation. We sought to (1) describe fluid balance patterns in ELGANs during the first 2 postnatal weeks, (2) evaluate the association of fluid balance with mechanical ventilation at postnatal day 14, and (3) investigate the association of fluid balance with an important clinical outcome, BPD. Our primary hypothesis was that a more positive fluid balance during the first 2 postnatal weeks would be associated with mechanical ventilation on postnatal day 14.

## Methods

### Study Population

The PENUT trial was a phase 3, placebo-controlled, intention-to-treat randomized clinical trial of erythropoietin in ELGANs conducted in 19 academic centers and 30 neonatal intensive care units in the US from December 1, 2013, to September 31, 2016.^15^ The inclusion criteria were (1) gestational age between 24 0/7 weeks and 27 6/7 weeks, (2) enrollment at less than 24 hours of age, and (3) arterial or venous access. Exclusion criteria included (1) major life-threatening anomalies, (2) hematologic crises (eg, disseminated intravascular coagulation and hemolysis), (3) hematocrit higher than 65% (to convert to a proportion of 1.0, multiply by 0.01), (4) hydrops fetalis, and (5) congenital infection. For this secondary analysis, we excluded neonates who died in the first 14 days. Participant race and ethnicity were determined by maternal self-report as part of the original PENUT study.^15^ These characteristics were assessed as part of the parent study, which as a randomized clinical trial sought to achieve balance in groups in regard to multiple factors, including race and ethnicity. The institutional review board at the University of Washington served as the central institutional review board, and each center involved in the study received approval from their institutional review boards or human research ethics committees. Informed written consent was obtained from the parent or legal guardian. This post hoc analysis was performed in November 2021 and followed the Strengthening the Reporting of Observational Studies in Epidemiology (STROBE) reporting guideline for observational studies.^16^

### Fluid Balance Definitions

The PENUT trial protocol (Supplement 1) included daily weights, total fluid intake, and outputs for the first 14 postnatal days, when available. We used daily weights to define fluid balance based on recent consensus guidelines and to be consistent with previous studies,^1,7,17^ because daily weights are more reliably recorded than complete input and output data. The use of successive weights is an excellent approximation of net fluid balance and is the recommended method of assessing fluid balance in this patient population.^1,2,18^

Fluid balance was calculated by comparing daily weight with birth weight:

Fluid Balance = [(Daily Weight − Birth Weight) / Birth Weight] × 100.

We evaluated fluid balance over the first 2 postnatal weeks in 5 different ways, including the maximum percentage of weight gain (peak positive fluid balance), the maximum percentage of weight loss (peak negative fluid balance), percentage of weight change at postnatal day 3, percentage of weight change at postnatal day 7, and the day the neonate returned to birth weight.^1^

### Acute Kidney Injury and Covariate Definition

The PENUT trial extracted all serum creatinine measurements during the study period. Acute kidney injury (AKI) was defined using the neonatal modified Kidney Disease: Improving Global Outcomes (KDIGO) serum creatinine criteria only, consistent with previous PENUT publications.^13,14,15^ Stage 1 is defined as a 1.5- to 1.9-times increase or an increase of 0.3 within 48 hours, stage 2 is a 2.0- to 2.9-times increase, and stage 3 is a greater than 3-times increase from baseline or serum creatinine greater than 2.5 mg/dL (to convert to micromoles per liter, multiply by 88.4). Acute kidney injury was adjudicated using the lowest prior creatinine level (before AKI) as the baseline, and a creatinine level had to surpass a threshold of at least 0.5 mg/dL for diagnosis. Assessment of AKI began on postnatal day 3.^13^ Severe AKI was defined as KDIGO stage 2 or 3. Other included covariates included all stages of intraventricular hemorrhage classified according to Papile grade and any stage of necrotizing enterocolitis classified by Bell stage.^15^

### Outcomes

The primary outcome was invasive mechanical ventilation (high-frequency or conventional ventilation) on postnatal day 14. Our secondary outcome was severe BPD or death according to Neonatal Research Network definitions by Jensen criteria.^19^ We determined BPD severity by respiratory support at 36 weeks’ postmenstrual age and severe BPD as receipt of 30% oxygen or more or positive pressure at 36 weeks’ postmenstrual age. We used a composite of severe BPD and death between day 14 and 36 weeks’ postmenstrual age as our secondary outcome.^12,18^

### Statistical Analysis

Categorical variables were analyzed by proportional differences with the χ^2^ test or Fisher exact test. A 2-tailed, unpaired *t* test and Wilcoxon rank sum test were used to compare continuous and ordinal variables, respectively. Odds ratios (ORs) and associated 95% CIs for the association between fluid balance variables and outcomes of interest were estimated from unconditional logistic regression models. Multivariable logistic regression models, selected using a backward selection process for maximum fluid balance and based on variables with *P* < .05 in bivariate analysis, were used to account for potential confounding variables, and findings are reported as adjusted ORs. The same model covariates were included in the subsequent models for minimum fluid balance, maximum fluid balance in the first 3 days of life, and maximum fluid balance in the first 7 days of life to make the associations comparable. We performed a sensitivity analysis and best-case/worst-case analysis to assess excluded infants. In all analyses, a 2-tailed *P* < .05 was considered statistically significant. Analysis was performed using SAS software, version 9.4 (SAS Institute Inc).

## Results

### Patient Characteristics

Of the 941 neonates who met inclusion criteria for PENUT, 923 neonates were eligible for analysis and 874 were included in our analysis (eFigure 1 in Supplement 2). The mean (SD) birth weight was 801 (188) g; 449 (51.4%) neonates were male, and 425 (48.6%) were female; 187 (21.4%) were of Hispanic maternal ethnicity, 676 (77.3%) were non-Hispanic, and 11 (1.3%) had unknown ethnicity; and 226 (25.9%) were Black, 569 (65.1%) were White, 51 (5.8%) were of other race, and 28 (3.0%) were of unknown race. Common maternal comorbidities included multiple gestation pregnancy (228 [24.7%]), chronic hypertension (66 [7.6%]), and diabetes (47 [5.8%]). Acute kidney injury occurred in 336 neonates (39.4%), and 162 (18.5%) had severe AKI (stage 2/3). Neonatal complications were common, including necrotizing enterocolitis (91 [10.4%]), patent ductus arteriosus (371 [41.5%]), and intraventricular hemorrhage (317 [36.4%]). A total of 577 neonates (66.0%) in the cohort had BPD, including 291 (33.3%) with severe BPD or death (Table 1).

**Table 1.** Comparison of Maternal and Neonatal Characteristics by Mechanical Ventilation Status on Postnatal Day 14^a^

Characteristic	Total cohort (N = 874)	Mechanical ventilation at 14 d (n = 458)	No mechanical ventilation at 14 d (n = 416)	*P* value^a^
Sex				
Male	449 (51.4)	245 (53.5)	203 (48.9)	.18
Female	425 (48.6)	213 (46.5)	212 (51.1)	
Maternal ethnicity				
Hispanic	187 (21.4)	84 (18.3)	103 (24.8)	.01
Non-Hispanic	676 (77.3)	365 (79.7)	310 (74.7)	
Unknown	11 (1.3)	9 (2.0)	2 (0.5)	
Maternal race				
Black	226 (25.9)	130 (28.4)	97 (23.4)	.07
White	569 (65.1)	283 (61.8)	285 (68.7)	
Other	51 (5.8)	26 (5.7)	25 (6.0)	
Unknown	28 (3.2)	19 (4.1)	8 (1.9)	
Birth weight, mean (SD), g	801 (188)	729 (163)	893 (174)	<.001
Birth length, mean (SD), cm	32.9 (2.9)	31.9 (2.7)	34.2 (2.5)	<.001
Gestational age, wk				
24	207 (23.7)	181 (39.5)	26 (6.3)	<.001
25	226 (25.8)	147 (32.1)	79 (19.0)	
26	212 (24.3)	81 (17.7)	130 (31.3)	
27	229 (26.2)	49 (10.7)	180 (43.4)	
Small for gestational age^b^	141 (16.3)	80 (17.5)	50 (12.1)	.03
Apgar score, median (IQR)^c^				
1 Minute	4 (2-6)	3 (1-5)	5 (3-6)	<.001
5 Minutes^c^	7 (5-8)	6 (4-7)	7 (6-8)	<.001
Maternal characteristics				
Multiple gestations	228 (26.1)	116 (25.3)	112 (27.0)	.58
Hypertension	66 (7.6)	40 (8.7)	26 (6.3)	.17
Diabetes	47 (5.4)	21 (4.6)	26 (6.3)	.27
Mode of delivery				
Cesarean section				
Scheduled	75 (8.6)	42 (9.2)	33 (8.0)	.22
Unscheduled	526 (60.2)	285 (62.2)	241 (58.0)	
Vaginal birth	273 (31.2)	131 (28.6)	141 (34.0)	
Prenatal steroid doses				
1	166 (21.2)	86 (21.1)	80 (21.3)	.01
2	545 (69.6)	296 (72.7)	248 (66.1)	
3	72 (9.2)	25 (6.1)	47 (12.5)	
Vasopressor use	268 (30.7)	212 (46.3)	56 (13.5)	<.001
Neonatal course				
Necrotizing enterocolitis	91 (10.4)	55 (12.0)	36 (8.7)	.11
Treated patent ductus arteriosus	371 (42.4)	271 (59.2)	99 (23.9)	<.001
Intraventricular hemorrhage	317 (36.3)	218 (47.6)	98 (23.7)	<.001
AKI	336 (38.4)	235 (51.3)	101 (25.5)	<.001
Severe AKI	162 (18.5)	126 (27.5)	36 (8.7)	<.001
BPD	577 (66.0)	380 (83.0)	197 (47.5)	<.001
Severe BPD	291 (33.3)	219 (47.8)	72 (17.3)	<.001
Treatment with erythropoietin	432 (49.4)	196 (47.8)	236 (51.5)	.20

Abbreviations: AKI, acute kidney injury; BPD, bronchopulmonary dysplasia.

^a^ Data are presented as number (percentage) of patients unless otherwise indicated.

^b^ Data available in 865 individuals.

^c^ Estimated from χ^2^ and Wilcoxon rank sum test for categorical and continuous variables, respectively.

### Fluid Balance

A total of 13 845 potential patient-days occurred during the first 2 postnatal weeks for the entire population. Weight was recorded on 13 388 possible patient-days (96.7%). Although 13 568 potential patient-days (98.0%) of intake data were available, complete intake and output data were available on only 1702 patient-days (12.3%).

Fluid balance in the first 2 postnatal weeks is given in Table 2. Median peak positive fluid balance was 11% (IQR, 4%-20%), occurring on postnatal day 13 (IQR, 9-14). Ninety-three neonates (10.6%) never decreased below their birth weight. Peak positive fluid balance was higher in neonates who died (18%; IQR, 10%-24%) (death between postnatal day 14 and 36 weeks’ postmenstrual age) than in those who survived (11%; IQR, 4%-19%; *P* = .001). The median peak negative fluid balance during the first 2 postnatal weeks was 10% below birth weight (IQR, −15% to −6%) and occurred on postnatal day 3 (IQR, 2-5). At postnatal day 3, median fluid balance was 7% below birth weight (IQR, −12% to 0%), and at postnatal day 7, median fluid balance was 3% below birth weight (IQR, −8% to −4%). Median fluid balance is shown in eFigure 2A in Supplement 2 and by gestational age in eFigure 2B in Supplement 2. There was a significant difference in the distribution of peak fluid balance between trial sites; however, trial site only explains 0.8% of the variation in peak fluid balance (eFigure 3 in Supplement 2).

**Table 2.** Fluid Balance Stratified by Mechanical Ventilation on Postnatal Day 14^a^

Fluid exposure	Median (IQR), %			*P* value
	Total cohort (N = 874)	Postnatal day 14 mechanical ventilation		
		Yes (n = 458)	No (n = 416)	
Fluid balance in the first 14 d				
Peak	11 (4 to 20)	15 (8 to 24)	8 (2 to 14)	<.001
Lowest	−10 (−15 to −6)	−10 (−15 to −5)	−11 (−15 to −7)	.04
Postnatal fluid balance at				
Day 3	−7 (−12 to 0)	−5 (−11 to 0)	−8 (−12 to −3)	<.001
Day 7	−3 (−8 to −4)	−4 (−9 to 2)	−1 (−7 to 7)	<.001
Days to regain birth weight	6 (8 to 10)	7 (5 to 10)	8 (6 to 11)	<.001

^a^ Estimated from a Wilcoxon rank sum test.

### Association of Fluid Balance With Outcomes

#### Primary Outcome: Mechanical Ventilation

A total of 458 neonates (52.4%) received mechanical ventilation on postnatal day 14 (Table 1). Those who required mechanical ventilation on postnatal day 14 were more likely to be born at lower gestational age and birth weights, have lower Apgar scores, receive vasopressors, and have an episode of AKI (Table 1; eTable 1 in Supplement 2).

Mechanical ventilation at postnatal day 14 was associated with fluid balance on postnatal days 3 and 7, peak positive fluid balance, and day at which neonates regained their birth weight (Table 2). Neonates whose daily weights never decreased below birth weight were more likely to require mechanical ventilation on postnatal day 14 compared with those whose weight decreased below birth weight (63 [67.7%] vs 404 [51.8%], *P* = .004). Median peak positive fluid balance was higher in neonates requiring mechanical ventilation on postnatal day 14 compared with those not requiring mechanical ventilation (15% above birth weight vs 8% above birth weight, *P* < .001). Neonates requiring mechanical ventilation at postnatal day 14 were more likely to have a less negative fluid balance on day 3 (5% below birth weight vs 8% below birth weight, *P* < .001). We noted the converse at postnatal day 7, when neonates requiring mechanical ventilation at postnatal day 14 were more likely to have had a more negative fluid balance (4% below birth weight vs 1% below birth weight, *P* < .001).

Neonates requiring mechanical ventilation regained their birth weight 1 day earlier than those not requiring mechanical ventilation at day 14 (7 vs 8 days, *P* < .001) (Figure, A; eFigure 2A in Supplement 2). This finding was most notable in the smallest gestational age cohort, in which neonates requiring mechanical ventilation regained their birth weight by postnatal day 4 compared with those who did not require mechanical ventilation, who regained their birth weight by postnatal day 7 (eFigure 2A in Supplement 2).

**Figure.**
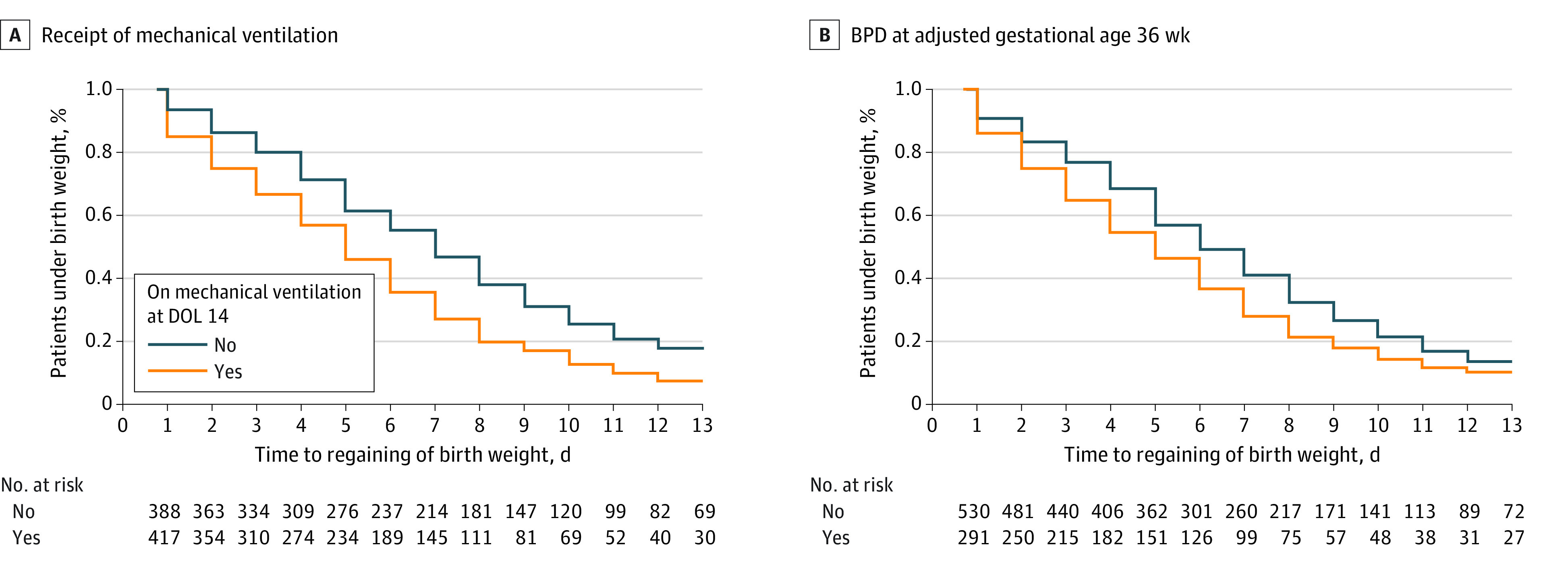
Kaplan-Meier Curve of Time to Regain Birth Weight BPD indicates bronchopulmonary dysplasia; DOL, day of life.

After adjusting for confounding variables, peak positive fluid balance during the first 14 postnatal days, fluid balance on postnatal day 3, and fluid balance on postnatal day 7 remained independently associated with the need for mechanical ventilation on postnatal day 14 (Table 3). In multivariable analyses, for each 10% increase in peak positive fluid balance, there was 103% increased odds of mechanical ventilation at postnatal day 14 (adjusted OR, 2.03; 95% CI, 1.64-2.51). Similarly, for every 10% increase in the fluid balance at postnatal day 3, there was 66% increased odds of mechanical ventilation at postnatal day 14 (adjusted OR, 1.66; 95% CI, 1.30-2.14). These findings remained significant in our best-case/worst-case analysis of excluded infants.

**Table 3.** Crude and Adjusted ORs and Associated 95% CIs for the Association Between Each 10% Increase in Fluid Balance and Mechanical Ventilation on Postnatal Day 14^a^

Exposure of interest	OR (95% CI)		*P* value
	Crude	Adjusted^b^	
Fluid balance			
Peak	2.09 (1.79-2.45)	2.03 (1.64-2.51)	<.001
Lowest	0.83 (0.67-1.03)	0.67 (0.50-0.91)	.01
Postnatal fluid balance			
Day 3	1.62 (1.36-1.94)	1.66 (1.30-2.14)	<.001
Day 7	1.43 (1.23-1.67)	1.41 (1.15-1.74)	<.001

Abbreviation: OR, odds ratio.

^a^ Estimated from unconditional logistic regression models.

^b^ Adjusted for gestational age, 5-minute Apgar score, treated patent ductus arteriosus, intraventricular hemorrhage, study site, acute kidney injury in the first 2 weeks of life, and vasopressor use.

Neonates with peak positive fluid balance more than 5% above their birth weight had 75% increased odds of mechanical ventilation at postnatal day 14 (adjusted OR, 1.75; 95% CI, 1.33-2.31). Neonates with peak positive fluid balance greater than 10% had similar odds of requiring mechanical ventilation on day 14 despite a doubling of the degree of fluid overload (adjusted OR, 1.74; 95% CI, 1.41-2.15) (Table 4).

**Table 4.** ORs and Associated 95% CIs for the Association Between Fluid Balance Threshold and Mechanical Ventilation and Severe BPD or Death by Fluid Balance^a^

Fluid balance	OR (95% CI)	*P* value
Ventilation on postnatal day 14		
>5%	1.75 (1.33-2.31)	<.001
>10%	1.74 (1.41-2.15)	<.001
>16%	1.75 (1.55-1.98)	<.001
Severe BPD or death		
>5%	1.51 (1.11-2.06)	.009
>10%	1.58 (1.28-1.96)	<.001
>16%	1.64 (1.36-1.97)	<.001

Abbreviations: BPD, bronchopulmonary dysplasia; OR, odds ratio.

^a^ Estimated from general estimating equation logistic regression models.

#### Fluid Balance and BPD or Death

A total of 291 neonates (33.3%) had severe BPD or died (after 14 days but before 36 weeks’ postmenstrual age) (eTables 2 and 3 in Supplement 2). Severe BPD or death was associated with peak positive fluid balance (median, 15% [IQR, 8%-23%] above their birth weight vs 9% [IQR, 3%-16%] above their birth weight among those with mild or no BPD; *P* < .001). In addition, neonates with severe BPD or who had died had regained their birth weight 1 day earlier than those with mild or no BPD (median, 7 [IQR, 5-9] days vs 8 [IQR, 6-10] days; *P* < .001) (Figure, B; eTable 3 in Supplement 2).

Neonates with peak positive fluid balance more than 5% above their birth weight had 51% increased odds of severe BPD or death (adjusted OR, 1.51; 95% CI, 1.11-2.06). Neonates with peak positive fluid balance more than 10% above their birth weight had 58% increased odds of severe BPD or death (adjusted OR, 1.58; 95% CI, 1.28-1.96). A peak positive fluid balance greater than 16% was most strongly associated with severe BPD or death (adjusted OR, 1.64; 95% CI, 1.36-1.97) (Table 4). In multivariable analyses for every 10% increase in peak positive fluid balance, there was no significant change in the odds of severe BPD or death (adjusted OR, 1.00; 95% CI, 0.95-1.05) (eTable 4 in Supplement 2).

## Discussion

In this secondary analysis of a large, prospective, multicenter, placebo-controlled randomized clinical trial of extremely premature neonates, we report the epidemiology of fluid balance in the first 2 postnatal weeks and its association with respiratory outcomes. We found that fluid balance in the first 2 postnatal weeks is independently associated with the need for mechanical ventilation at postnatal day 14 as well as severe BPD or death. Fluid balance on postnatal day 3 is associated with the need for mechanical ventilation at postnatal day 14 as well as the development of BPD. Furthermore, time to regain birth weight may be an important indicator of short- and long-term pulmonary complications.

Studies^1,2,10^ on the impact of fluid balance in critically ill premature neonates to date have focused on fluid balance during the first week and almost exclusively on short-term outcomes and mortality. Our findings are consistent with previous work^3,4,5,6^ in critically ill pediatric patients showing that fluid balance is associated with respiratory outcomes. Recent work^1,2^ in neonates born at less than 29 weeks’ gestational age from the AWAKEN cohort found that positive fluid balance during the first postnatal week was associated with the need for mechanical ventilation on postnatal day 7, and a negative fluid balance on postnatal day 7 was associated with a decreased risk of mechanical ventilation. In a recent publication^20^ in the PENUT cohort, higher total fluid intake in the first postnatal week was associated with an increased risk of patent ductus arteriosis and necrotizing enterocolitis. However, the authors did not find an association between their exposures and severe BPD and did not evaluate mechanical ventilation as an outcome. The current study extends these findings by showing an association between fluid balance and outcomes in preterm neonates over a longer postnatal period, identifying fluid balance on postnatal day 3 and time to regain birth weight as potential clinical targets warranting further study. We note that, in univariable analysis, peak fluid balance and fluid balance on postnatal day 3 were associated with development of BPD. Furthermore, our findings suggest for the first time that 5% fluid overload in the first 2 weeks may be clinically significant in premature neonates because those with even small degrees of fluid overload are at increased risk of long-term pulmonary morbidity.

Although fluid overload of more than 10% is considered pathologic in older children and adults, the thresholds of fluid overload should be systematically reevaluated in premature newborns given their unique physiology. Premature neonates are born with an excess of extracellular fluid and undergo a diuresis during the first several days following birth with an expected decrease in body weight. The loss of extracellular fluid is crucial in the transition to extrauterine life and is accomplished by natriuresis and a negative fluid balance. The current study provides novel epidemiologic data that suggest that 5% fluid overload during the first 14 postnatal days may represent an important threshold in the management of critically ill premature neonates. We also found that neonates who do not lose weight after birth have an increased risk of poor respiratory outcomes. Additionally, the day at which premature neonates regain birth weight may be an important indicator of clinical fluid balance. Further work should focus on defining gestational age–specific fluid balance thresholds and development of fluid balance and weight curves in prospective cohorts to better understand the contribution of fluid balance to short- and long-term respiratory outcomes.

Previous work^1,2,11^ in premature neonates has recognized that higher fluid balance in the early postnatal period is associated with the development of BPD. In a secondary analysis^11^ of the Trial of Indomethacin Prophylaxis in Preterms, which evaluated the efficacy of indomethacin in premature neonates, those randomized to receive indomethacin had reduced weight loss and higher rates of BPD. The authors postulated that decreased urine output, a known adverse effect of indomethacin, may have resulted in a positive fluid balance and an increased BPD incidence. This pathophysiologic mechanism may in part explain recent findings^12^ that premature neonates with AKI are more likely to develop BPD.

### Strengths and Limitations

This study has several strengths. One strength is the robust, high-quality data available from a large, prospective multicenter study,^15^ allowing for the detailed exploration of the impact of fluid balance on respiratory outcomes in ELGANs. Our results confirm the results of these previous studies^1,2^ because we found that neonates who had a higher peak fluid balance had increased odds of needing mechanical ventilation on postnatal day 14. More importantly, on adjusted analysis, a fluid balance greater than 5% increases the odds of mechanical ventilation by almost 2-fold. These data suggest that it may be beneficial to focus on preventing a positive fluid balance greater than 5% to improve outcomes. More importantly, even small degrees of positive fluid balance may increase morbidity and mortality in ELGANs.

There are also several limitations of this secondary analysis. Our findings are limited to only clinically available weights and laboratory values, which were not protocolized. We note that weights were available for 97% of all possible patient-days; therefore, misclassification is likely to have been infrequent. Although we excluded infants who died before 14 days, a sensitivity analysis of these excluded infants did not change our conclusions. Despite efforts to include all factors, residual confounding may remain in this analysis. Our findings are associations due to study design, and fluid balance may be in part a proxy of critical illness in premature neonates. We also note that there are other variables, such as clinical conditions and clinical practices, that may result in changes in weight and are not captured in this analysis. Because the PENUT database included only daily weights from the first 2 postnatal weeks, our conclusions were limited to this period. Longer observation periods are necessary to extend our findings and identify other potential critical indicators of fluid balance associated with outcomes.

## Conclusions

In this multicenter, retrospective secondary analysis of a randomized clinical trial, we describe the distribution and impact of fluid balance in preterm neonates over the first 2 postnatal weeks. Our study found that peak positive fluid balance during the first 2 postnatal weeks and fluid balance on postnatal days 3 and 7 were independently associated with mechanical ventilation on postnatal day 14 and suggests that time to return to birth weight and fluid overload greater than 5% in the first 2 postnatal weeks are clinically important markers of fluid status. Future prospective studies should evaluate the impact of fluid management strategies on subsequent short- and longer-term respiratory outcomes for premature neonates.

## Notes

Supplement 1.
Trial Protocol

Supplement 2. **eTable 1.** Mechanical Ventilation by Gestational Age **eTable 2.** Comparison of Maternal and Neonatal Characteristics by Severe BPD/Death Status **eTable 3.** Median Fluid Balance Stratified by Severe Bronchopulmonary Dysplasia (BPD)/Death Status **eTable 4.** Crude and Adjusted Odds Ratios (ORs) and Associated 95% Confidence Intervals (CIs) for the Association Between Each 10% Increase in Fluid Balance and Severe Bronchopulmonary Dysplasia (BPD) or Death **eFigure 1.** Consort Diagram **eFigure 2.** Association of Fluid Balance and Mechanical Ventilation on Postnatal Day 14 (A) Among Entire Cohort and (B) Stratified by Gestational Age **eFigure 3.** Box Plot of Peak Fluid Balance by Study Site

Supplement 3.
Neonatal Kidney Collaborative Research Committee

Supplement 4.
Data Sharing Statement
