# Supplementary material for: Association of Fluid Balance With Short- and Long-term Respiratory Outcomes in Extremely Premature Neonates: A Secondary Analysis of a Randomized Clinical Trial
Source: JAMA Netw Open. 2022 Dec 29;5(12):e2248826. doi: 10.1001/jamanetworkopen.2022.48826 (PMC9856967; doi:10.1001/jamanetworkopen.2022.48826)
Supplement: Supplement 2. — eTable 1. Mechanical Ventilation by Gestational Age eTable 2. Comparison of Maternal and Neonatal Characteristics by Severe BPD/Death Status eTable 3. Median Fluid Balance Stratified by Severe Bronchopulmonary Dysplasia (BPD)/Death Status eTable 4. Crude and Adjusted Odds Ratios (ORs) and Associated 95% Confidence Intervals (CIs) for the Association Between Each 10% Increase in Fluid Balance and Severe Bronchopulmonary Dysplasia (BPD) or Death eFigure 1. Consort Diagram eFigure 2. Association of Fluid Balance and Mechanical Ventilation on Postnatal Day 14 (A) Among Entire Cohort and (B) Stratified by Gestational Age eFigure 3. Box Plot of Peak Fluid Balance by Study Site [file jamanetwopen-e2248826-s002.pdf]

Supplementary Online Content

Starr MC, Griffin R, Gist KM, et al; Neonatal Kidney Collaborative Research Committee. Association of fluid balance with short- and long-term respiratory outcomes in extremely premature neonates: a secondary analysis of a randomized clinical trial. *JAMA Netw Open*. 2022;5(12):e2248826. doi:10.1001/jamanetworkopen.2022.48826

eTable 1. Mechanical Ventilation by Gestational Age
eTable 2. Comparison of Maternal and Neonatal Characteristics by Severe BPD/Death Status
eTable 3. Median Fluid Balance Stratified by Severe Bronchopulmonary Dysplasia (BPD)/Death Status
eTable 4. Crude* and Adjusted† Odds Ratios (ORs) and Associated 95% Confidence Intervals (CIs) for the Association Between Each 10% Increase in Fluid Balance and Severe Bronchopulmonary dysplasia (BPD) or Death
eFigure 1. Consort Diagram
eFigure 2. Association of Fluid Balance and Mechanical Ventilation on Postnatal Day 14 (A) Among Entire Cohort and (B) Stratified by Gestational Age
eFigure 3. Box Plot of Peak Fluid Balance by Study Site

This supplementary material has been provided by the authors to give readers additional information about their work.

© 2022 Starr MC et al. *JAMA Network Open.*

eTable 1. Mechanical Ventilation by Gestational Age

	Mechanically Ventilated at Postnatal 14		
	Yes (n=458) No (%)	No (n=415) No (%)	p-value*
Gestational Age, n (%)			<0.001
24 weeks	181(39.5)	26(6.3)	
25 weeks	147(32.1)	79(19.0)	
26 weeks	81(17.7)	130(31.3)	
27 weeks	49(10.7)	180(43.4)	

* Estimated from a chi-square test

© 2022 Starr MC et al. *JAMA Network Open.*

**eTable 2.** Comparison of maternal and neonatal characteristics by severe BPD/Death Status			
	Severe BPD or Death (N=292) No (%)	No BPD or Mild BPD (N=582) No (%)	p-value
Male, n (%)	169 (57.9)	280(48.1)	0.006
Maternal Ethnicity, n (%)			0.82
Hispanic	59 (20.2)	128(22.0)	
Non-Hispanic	229 (78.4)	447(76.8)	
Unknown	4 (1.4)	7(1.20)	
Maternal Race, n (%)			0.92
White	193 (66.1)	376(64.6)	
Black	73 (25.0)	154(26.5)	
Other	16 (5.5)	35(6.0)	
Unknown	10 (3.4)	35(6.0)	
Birth weight, g, mean (SD)	743 (178)	840 (183)	<0.001
Birth length, cm, mean (SD)	32.0 (2.8)	33.5 (2.8)	<0.001
Gestational Age, n (%)			<0.001
24 weeks	89 (30.5)	118(20.3)	
25 weeks	84 (28.8)	142(24.4)	
26 weeks	61 (20.9)	151(30.0)	
27 weeks	58 (19.9)	171(29.4)	
Small for GA, n (%)	64 (22.0)	66(11.4)	<0.001
Median (IQR) Apgar 1 Minute*	3(1,5)	4(2,6)	<0.001
Median (IQR) Apgar 5 Minutes*	6(5,7)	7(6,8)	<0.001
**Maternal characteristics**			
Multiple gestations	93(27.3)	150(25.8)	0.62
Hypertension	28(8.2)	42(7.2)	0.58
Diabetes	21(6.2)	27(4.6)	0.32
Mode of Delivery, n (%)			0.003
C-Section (Scheduled)	199 (68.2)	327(56.2)	
C-Section (Unscheduled)	18 (6.2)	57(9.8)	
Vaginal Birth	75 (25.7)	198(34.0)	
Prenatal steroids, n (%)			0.14
1 dose	48 (18.0)	118(22.9)	
2 doses	189 (70.8)	356(69.0)	
3 doses	30 (11.2)	42(8.1)	
Vasopressor use, n (%)	123 (42.1)	146 (25.1)	<0.001
**Neonatal Course**			
Necrotizing Enterocolitis, n (%)	42 (14.4)	49(8.4)	0.006
Patent Ductus Arteriosus, n (%)	154 (52.7)	217(37.4)	<0.001
Intraventricular Hemorrhage, n (%)	127 (43.8)	190(32.7)	0.001
AKI	139 (48.1)	197(34.9)	<0.001
* Estimated from a chi-square and Wilcoxon rank sums test for categorical and continuous variables, respectively			

© 2022 Starr MC et al. *JAMA Network Open.*

eTable 3. Median Fluid Balance stratified by Severe bronchopulmonary dysplasia (BPD)/Death Status

	Severe BPD or Death (N=292) Median (IQR)	No BPD or Mild BPD (N=582) Median (IQR)	p-Value*
Peak Fluid Balance First 14 d	15% (8%, 23%)	9% (3%, 16%)	<0.001
Lowest Fluid Balance First 14 d	-10% (-15%, -6%)	-10% (-15%, -6%)	0.40
Fluid Balance at Postnatal Day 3	-5% (-11%, 0%)	-8% (-12%, -1%)	0.005
Fluid Balance at Postnatal Day 7	-2% (-7%, 5%)	-3% (-9%, 4%)	0.017
Day to regain birthweight	7 (5, 9)	8 (6, 10)	<0.001

* Estimated from a Wilcoxon rank sums test

© 2022 Starr MC et al. *JAMA Network Open.*

eTable 4. Crude* and Adjusted† odds ratios (ORs) and associated 95% confidence intervals (CIs) for the association between each 10% increase in fluid balance and Severe Bronchopulmonary dysplasia (BPD) or Death

Exposure of Interest	Crude (OR, 95% CI)	Adjusted (OR, 95% CI)	Adjusted with steroids (OR, 95% CI)	p-value
Peak fluid balance (per 10% increase)	1.04 (0.96-1.13)	1.00 (0.96-1.05)	1.00 (0.95-1.05)	1.00
Lowest fluid balance (per 10% decrease)	0.82 (0.6-1.04)	0.95 (0.73-1.23)	0.99 (0.75-1.30)	0.92
Fluid balance postnatal day 3 (per 10% increase)	1.37 (1.16-1.61)	1.13 (0.95-1.35)	1.13 (0.93-1.37)	0.21
Fluid balance postnatal day 7 (per 10% increase)	1.00 (0.96-1.04)	0.97 (0.88-1.08)	0.96 (0.84-1.09)	0.55
Day to regain birthweight (per 7 days)	0.47 (0.32-0.68)	0.66 (0.42-1.05)	0.66 (0.42-1.05)	0.08

*Estimated from unconditional logistic regression models adjusted for study site
†Adjusted for sex, gestational age, small size for gestational age, 5-minute Apgar, delivery method, study site, prenatal steroids, NEC, PDA, IVH, AKI in the first two weeks of life, and vasopressor use

© 2022 Starr MC et al. *JAMA Network Open.*

eFigure 1. Consort Diagram

941 subjects were enrolled in the PENUT study. We excluded five infants who were removed from the parent study (4 died prior to receiving study drug, and one who was enrolled incorrectly). Furthermore, we also excluded 13 infants who died on days 0, 1 or 2. We further excluded 49 infants who died prior to 14 days of age. Therefore, 874 infants with sufficient data for analysis and survival to 14 days of age were included in this analysis.

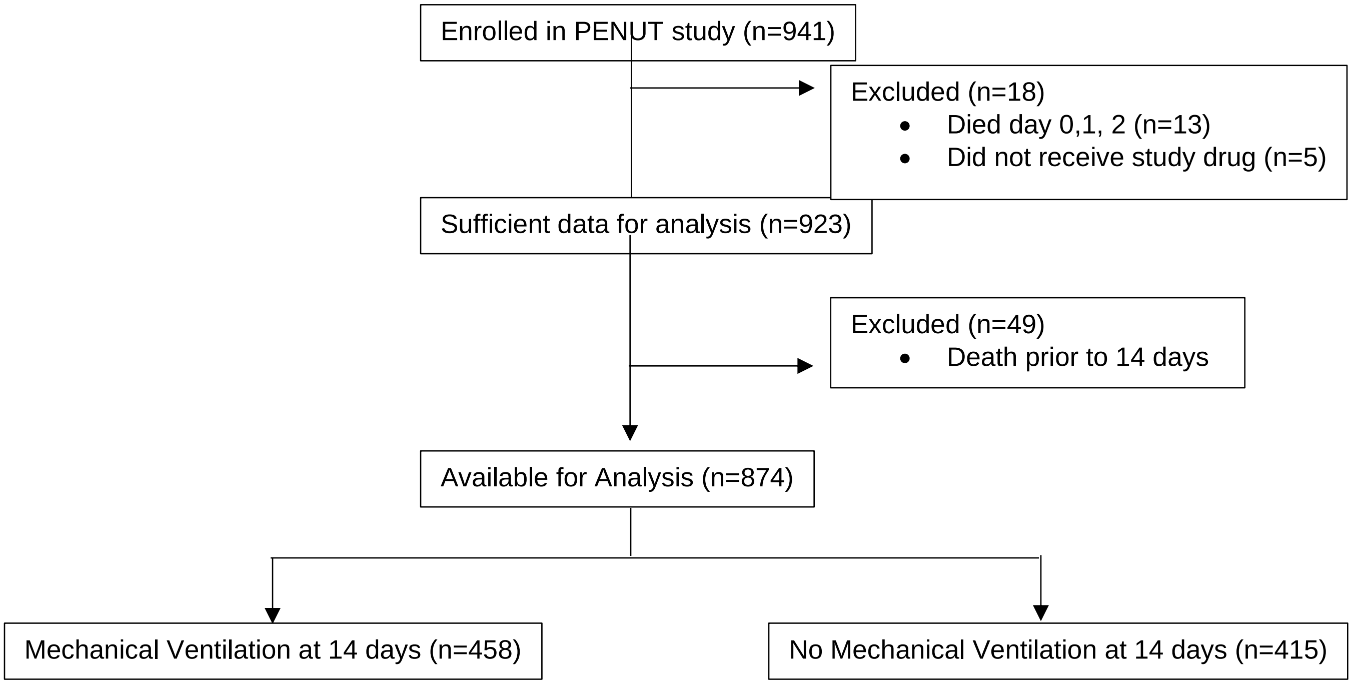

© 2022 Starr MC et al. *JAMA Network Open.*

eFigure 2. Association of Fluid Balance and Mechanical Ventilation on Postnatal Day 14 (A) Among Entire Cohort and (B) Stratified by Gestational Age

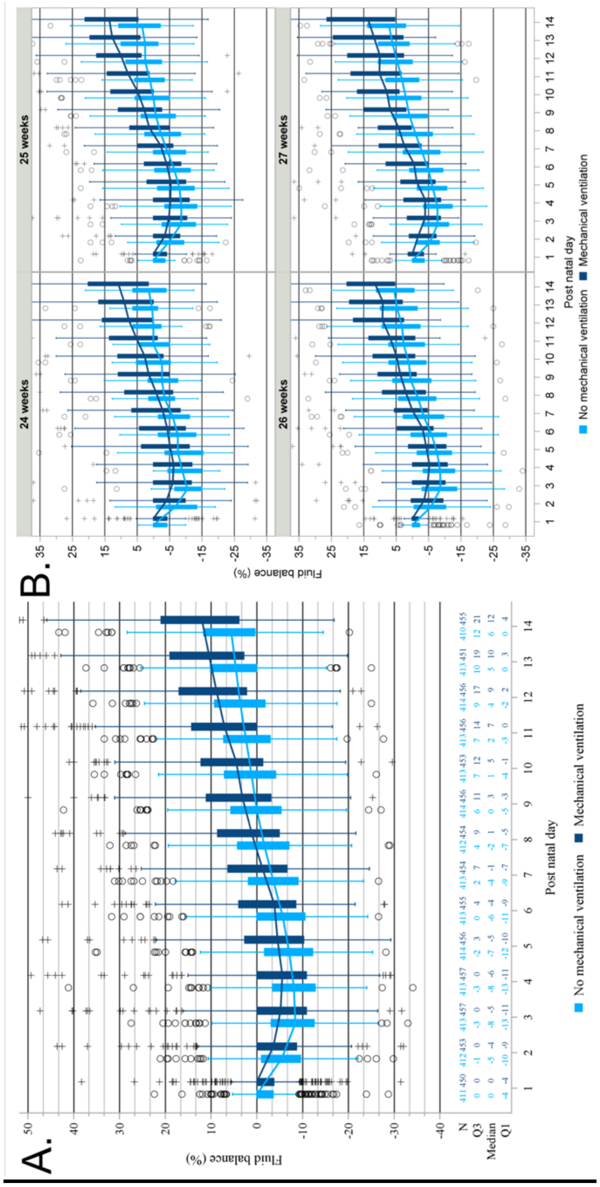

© 2022 Starr MC et al. *JAMA Network Open.*

eFigure 3. Box Plot of Peak Fluid Balance by Study Site

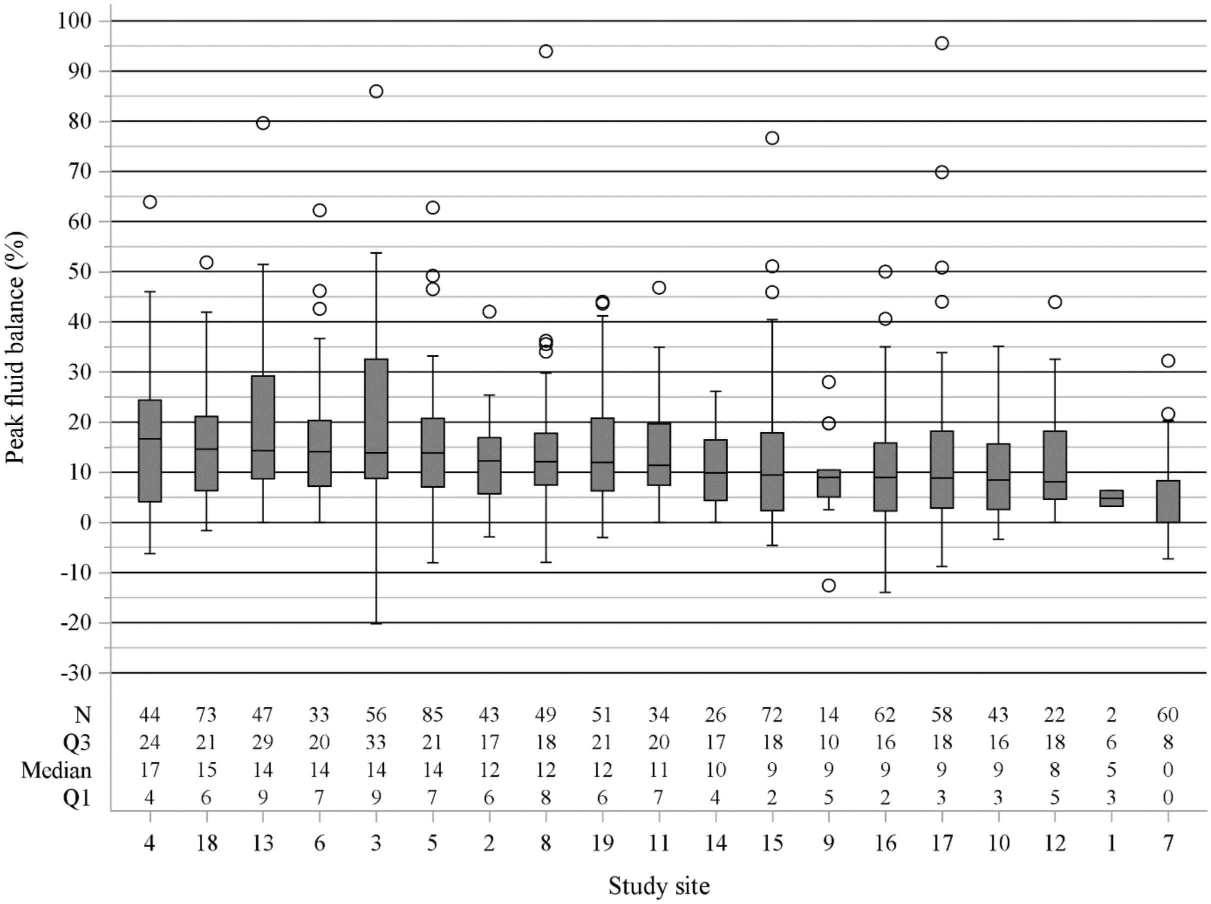

© 2022 Starr MC et al. *JAMA Network Open.*
