# Supplementary material for: Association of Fluid Balance With Short- and Long-term Respiratory Outcomes in Extremely Premature Neonates: A Secondary Analysis of a Randomized Clinical Trial
Source: JAMA Netw Open. 2022 Dec 29;5(12):e2248826. doi: 10.1001/jamanetworkopen.2022.48826 (PMC9856967; doi:10.1001/jamanetworkopen.2022.48826)
Supplement: Supplement 3. — Neonatal Kidney Collaborative Research Committee [file jamanetwopen-e2248826-s003.pdf]

Supplemental Online Content: Nonauthor Collaborators
*First name, last name, and suffix (if applicable) are required and will appear in PubMed.

***Group Name(s): Neonatal Kidney Collaborative Research Committee**							
***First Name and Middle Initial(s)**	***Last Name**	***Suffix (eg, Jr, III)**	Academic Degrees	Institution	Location (city, state/province, country)	Role or Contribution, eg, chair, principal investigator	Group (if more than 1 Group listed in the byline) and/or Subgroup (eg, Steering Committee)
Jennifer R	Charlton		MD, MSc	University of Virginia Children's Hospital	Charlottesville, Virginia, USA		
Marissa	DeFreitas		MD	Holtz Children's Hospital, University of Miami	Miami, Florida, USA		
Mamta	Fuloria		MBBS	Children's Hospital at Montefiore, Albert Einstein College of Medicine	Bronx, New York, USA.		
Mina	Hanna		MD	University of Kentucky	Lexington, Kentucky, USA.		
Matthew W.	Harer		MD	University of Wisconsin School of Medicine and Public Health	Madison, Wisconsin, USA.		
Cara	Slagle		MD	Cincinnati Children's Hospital Medical Center, University of Cincinnati College of Medicine	Cincinnati, Ohio, USA.		
Jonathan R.	Swanson		MD MSc	University of Virginia Children's Hospital	Charlottesville, Virginia, USA		
Keia	Sanderson		MD MSCR	University of North Carolina	Chapel Hill, North Carolina, USA.		
Meredith P.	Schuh		MD	Medical Center, University of	Cincinnati, Ohio, USA.		
Andrew M.	South		MD MS	Brenner Children's, Wake Forest University School of Medicine,	Winston Salem, North Carolina, USA.		
Heidi J.	Steflik		MD MSCR	Carolina, Charleston, South			
Robert	Woroniecki		MD	Stony Brook School of Medicine	Stony Brook, NY, USA.		
Michael	Zappitelli		MD MSc	University of Toronto, Toronto, Ontario, Canada			
