# Supplementary material for: Association of Fluid Balance With Short- and Long-term Respiratory Outcomes in Extremely Premature Neonates: A Secondary Analysis of a Randomized Clinical Trial
Source: JAMA Netw Open. 2022 Dec 29;5(12):e2248826. doi: 10.1001/jamanetworkopen.2022.48826 (PMC9856967; doi:10.1001/jamanetworkopen.2022.48826)
Supplement: Supplement 4. — Data Sharing Statement [file jamanetwopen-e2248826-s004.pdf]

Data Sharing Statement

Starr. Association of Fluid Balance With Short- and Long-term Respiratory Outcomes in Extremely Premature Neonates. *JAMA Netw Open*. Published December 29, 2022. doi:10.1001/jamanetworkopen.2022.48826

Data
Data available: Yes
Data types: Data dictionary, Deidentified participant data
How to access data: : De-identified individual participant data are available through the NINDS Data Archive: https://www.ninds.nih.gov/Current-;Research/Research- Funded-NINDS/Clinical-Research/Archived-Clinical- Research-Datasets. The data will be de-identified and a limited access data is available through a request form on that page. Data dictionaries, in addition to study protocol, the statistical analysis plan, and the informed consent form are included.
When available: beginning date: 01-01-2022

Supporting Documents
Document types: None

Additional Information
Who can access the data: The data will be made available to researchers who provide a methodologically sound proposal for use in achieving the goals of the approved proposal.
Types of analyses: All
Mechanisms of data availability: Through NINDS
